# An Equitable and Successful Antibiotic Stewardship Program Strategy to Prevent Clostridioides difficile Infection in Rural Communities

**DOI:** 10.1017/ash.2025.272

**Published:** 2025-09-24

**Authors:** Randee Mason, James Keegan

**Affiliations:** 1 Keegan Mason and Associates, LLC

## Abstract

Specialty Infectious Disease physician.

Clinical Associate Professor University of South Dakota Medical School

Consultants for South Dakota Department of Health regarding antibiotic stewardship

Consultants West Virginia Hospital Association regarding antibiotic stewardship **Background:** An equitable distribution of antibiotic stewardship expertise is a challenge for rural communities across the United States. The advantage rural communities have is that there are fewer barriers for implementation of effective antibiotic stewardship strategies.

The authors worked with several rural communities in the United States over the paste several years implementing a proven antibiotic stewardship strategy that has been shown to decrease Clostridioides infection. **Method:** Strategy employed was avoidance of the more common microbiome damaging broad spectrum antibiotics in favor of more targeted narrow spectrum antibiotics based on local antibiogram data. Additionally, ongoing infectious disease and antibiotic stewardship access for questions as well as data review with feedback were provided.

Findings: Clostrioides infection was eliminated in some communities and others markedly decreased as shown by a very low percentage of toxin positive, PCR positive to toxin negative PCR positive isolates expected for that region. **Conclusion:** This strategy is translatable to other communities accompanied by antibiotic stewardship expertise and support and can be a model for community wide antibiotic stewardship which further optimizes patient and resident safety from Clostidioides infection.

